# Correlation between Microstructure and Chemical Composition of Zinc Oxide Gas Sensor Layers and Their Gas-Sensitive Properties in Chlorine Atmosphere

**DOI:** 10.3390/s20236951

**Published:** 2020-12-05

**Authors:** Marta Fiedot-Toboła, Patrycja Suchorska-Woźniak, Kamila Startek, Olga Rac-Rumijowska, Rafał Szukiewicz, Monika Kwoka, Helena Teterycz

**Affiliations:** 1 Faculty of Microsystem Electronics and Photonics, Wrocław University of Science and Technology, Janiszewskiego 11/17, 50-372 Wrocław, Poland; patrycja.wozniak@pwr.edu.pl (P.S.-W.); olga.rac-rumijowska@pwr.edu.pl (O.R.-R.); helena.teterycz@pwr.edu.pl (H.T.); 2 Łukasiewicz Research Network—PORT Polish Center for Technology Development, Stabłowicka 147, 54-066 Wrocław, Poland; kamila.startek@port.lukasiewicz.gov.pl (K.S.); szuszu@ifd.uni.wroc.pl (R.S.); 3 Institute of Experimental Physics, University of Wroclaw, Maxa Borna 9, 50-204 Wroclaw, Poland; 4 Department of Cybernetics, Nanotechnology and Data Processing, Faculty of Automatic Control, Electronics and Computer Science, Silesian University of Technology, Akademicka 16, 44-100 Gliwice, Poland; monika.kwoka@polsl.pl

**Keywords:** resistive gas sensors, chlorine sensitivity, zinc oxide, microstructure, chemistry

## Abstract

In this article, we present results concerning the impact of structural and chemical properties of zinc oxide in various morphological forms and its gas-sensitive properties, tested in an atmosphere containing a very aggressive gas such as chlorine. The aim of this research was to understand the mechanism of chlorine detection using a resistive gas sensor with an active layer made of zinc oxide with a different structure and morphology. Two types of ZnO sensor layers obtained by two different technological methods were used in sensor construction. Their morphology, crystal structure, specific surface area, porosity, surface chemistry and structural defects were characterized, and then compared with gas-sensitive properties in a chlorine-containing atmosphere. To achieve this goal, scanning electron microscopy (SEM), X-ray diffraction (XRD), X-ray photoelectron spectroscopy (XPS) and photoluminescence spectroscopy (PL) methods were used. The sensing properties of obtained active layers were tested by the temperature stimulated conductance method (TSC). We have noticed that their response in a chlorine atmosphere is not determined by the size of the specific surface or porosity. The obtained results showed that the structural defects of ZnO crystals play the most important role in chlorine detection. We demonstrated that Cl_2_ adsorption is a concurrent process to oxygen adsorption. Both of them occur on the same active species (oxygen vacancies). Their concentration is higher on the side planes of the zinc oxide crystal than the others. Additionally, ZnO sublimation process plays an important role in the chlorine detection mechanism.

## 1. Introduction

Resistive gas sensors due to their simple construction, low cost and large range of detectable gases as carbon monoxide [1], nitrogen oxides [2] or methane [3], are widely used in commercial gas detection systems. Gas-sensitive materials used in such sensors are made of metal oxides with semiconductor properties. The electrical parameters of these oxides, such as work function or conductance, depends on the composition of the atmosphere being tested. Zinc oxide is the oldest known gas-sensitive material. In 1954, Heiland discovered that the resistance of zinc oxide depends on the composition of the atmosphere, which has become the basis for the development of sensors for determining the concentration of various organic substances such as methane (CH_4_) [4], propane (C _3_H_8_) [5], butane (C_4_H_10_) [6] or ethanol (C_2_H_5_OH) [7].

Despite very high sensitivity to some substances and the low cost of production, resistive gas sensors are characterized by a low selectivity. First attempts to eliminate this drawback were presented in 1967 by Shaver, who applied doping of metal oxides with noble metals such as Pt, Pd, Ir and Rh [8]. The process significantly improved the selectivity of these types of sensors and widened their area uses. However, despite a huge number of scientific studies conducted in this field, resistive gas sensors still exhibit an unsatisfactory selectivity [9,10,11,12,13]. Therefore, research directed at searching for new materials in order to eliminate this defect are still being carried out [14,15,16]. Among other things, gas-sensitive materials of various morphological forms and dimensions, undoped or catalytically doped with active nanoparticles, are synthesized and tested [17,18,19,20,21]. In addition to research focusing on material engineering, studies on the mechanism of interaction of various gases with gas-sensitive materials are very important. It is well known that the physicochemical processes that determine the sensor response occur at the gas oxide–semiconductor interface. For this reason, it is assumed that the basic parameters of the sensors, such as sensitivity, selectivity, response and recovery time, and stability are primarily determined by the specific surface area and porosity. These parameters are correlated and, according to literature reports, the most important one is the specific surface of the sensor material. It is widely believed that with the increase in the specific surface area of a material, the concentration of active centers increases, and therefore a greater number of atoms (or molecules) can participate in the physicochemical processes occurring at the gas–semiconductor interface. Among other things, this is why a lot of research concerns nanomaterials—they have the highest possible specific surface value. For this reason, sensors made of nanomaterials should have better sensitivity, selectivity, stability and shorter response and recovery times [21,22,23,24,25,26].

In the field of nanotechnology, a lot of research relates to different shapes of ZnO [27,28,29,30,31,32]. Due to the diversified structure of the ZnO crystallographic planes, this material creates the most diverse morphological forms of all known materials [33]. Choosing the appropriate method and parameters of synthesis, it crystallizes both in the nano-form as well as micrometric structures of such shapes as: rods [34,35], tubes [36], needles [37], spheres [38], cubes [39] and helix [40]. However, the preferable forms in which ZnO crystalizes are one-dimensional (1D) or quasi-one-dimensional (q-1D) structures, because during synthesis crystals of the lowest possible energy are formed [41,42,43]. Polar planes have much more surface energy than nonpolar planes. In conjunction with this, the crystal grows slower toward [01$\bar{1}$0] and $[2\bar{1}\bar{1}$0] and faster toward [0001] [44].

It is very important that, due to the anisotropic properties of each of the ZnO morphological forms, they have different physicochemical properties. Many authors indicated the correctness of this statement—for example, in relation to optical properties [30], catalytic [31] or biological [32] activity and gas sensitivity [45,46,47]. Taking into account the advantage of zinc oxide, wanting to choose the material for a specific application does not require searching for new compounds but needs to synthesize ZnO with the desired morphology.

Because of the many methods for synthesis, a very large ratio of surface to volume of material, 1D and/or q-1D ZnO structures are one of the most desirable forms of this semiconductor [48]. Chemical bath deposition (CBD) is a very popular method used to achieve this goal, because it is carried out at a low temperature, it does not require the use of complicated equipment and the change in process parameters allows modification of the microstructure of the obtained zinc oxide forms. According to literature reports, it is also possible to produce q-1D ZnO structures directly on ceramic substrates, and the gas-sensitive layers thus obtained are characterized by better parameters compared to their volume equivalents [49,50].

ZnO gas sensors were used for detection of many different organic [4,5,6] and inorganic [51,52] gases. One of the most dangerous gases for which detection is extremely important is chlorine. Chlorine is commonly used in the treatment process of water and in the chemical industry it is one of the basic substrates. This oxidizing gas has an unpleasant suffocating smell, it is a strong poison and a destructive agent in the natural environment. Due to the harmfulness of this element, yet at the same time due to the very wide area of its applications, constant monitoring is required of its concentrations. The human chlorine detection threshold is less than 1 ppm. When the concentration of it is great, the sense of smell is impaired and it gradually causes more and more health consequences, ranging from irritation to the respiratory system to suffocation in extreme cases [53,54].

In this article, we present the results of the impact of ZnO structural and chemical properties on its gas-sensitive properties in the atmosphere containing a very aggressive chlorine gas. The presented research results are a part of projects related to cooperation with the water industry—i.e., chlorine detection in an atmosphere with high humidity. The influence of the humidity was presented in previous work [55]. Two types of ZnO layers obtained with the different methods were tested. Their morphology, crystal structure, specific surface and porosity were determined, then were correlated with gas-sensitive properties in a chlorine-containing atmosphere. The preliminary results in this field have been presented in recent papers [56]. This issue is complex due to difficulties with chlorine detection, which has strongly oxidizing properties. There are very few publications describing this phenomenon and the mechanism is not fully understood due to divergent conclusions [45,46,47]. Therefore, the aim of this research part was to understand the mechanism of chlorine detection using a resistive gas sensor with an active layer made of zinc oxide with a different structure and morphology.

## 2. Materials and Methods

**Figure 1:**
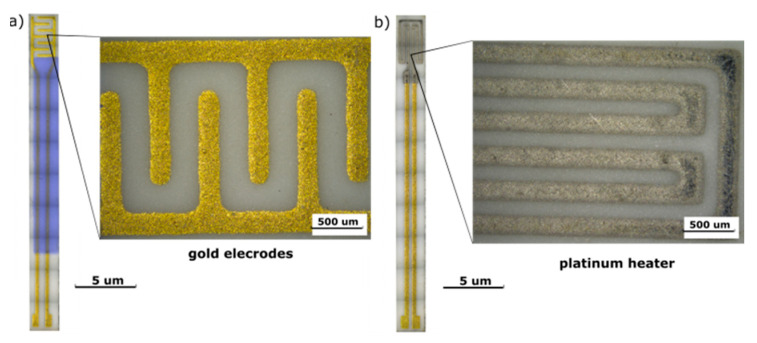
Images of the gas sensor surface from the side of: (**a**) gold electrodes and (**b**) platinum heater.

Gas-sensitive layers based on ZnO were made by thick-film technology and chemical bath deposition (CBD) method on a substrate with electrodes and a heater. The thickness of the alundum substrate (96% Al_2_O_3_) with dimensions of 25.40 × 2.45 mm^2^ was 250 µm. On one side of the substrate, there was a platinum heater in the form of a meander and gold contacts (ESL 8846-G, ESL Europe, Reading, UK). On the other side, gold electrodes and dielectric (ESL 4913-G, ESL Europe, Reading, UK) were printed as shown in the Figure 1.

The q-1D ZnO structures were deposited only on the side of the substrate on which the electrodes were located. The process was carried out with the chemical bath deposition method in an equimolar solution of aqueous zinc nitrate (Zn(NO_3_)_2_) and hexamethylenetetramine (HMT). In the first stage of process, a zinc nitrate solution and HMT solutions were prepared, each at a concentration of 1 M. Then, both solutions were mixed together in a 1:1 ratio and diluted; thus, the final concentration of individual compounds was 100 mM. The structure growth process was carried out at a temperature of 90 °C for 9 h at atmospheric pressure. After the process was completed, the sensor structures were rinsed in deionized water in an ultrasonic cleaner and then dried in an ambient atmosphere [57].

The ZnO powder was prepared by the precipitation of zinc chloride with sodium hydroxide. The reaction was carried out at 90 °C. The resulting precipitate was washed in deionized water, then dried at room temperature. In order to make the sensor layer, using the screen printing technique, a paste made of zinc oxide powder and organic carrier (ESL-403, ESL Europe, Reading, UK) was prepared. The paste was printed twice with a screen printer (DEK 1202, DEK, Munich, Germany) by drying the layers after each printing, first at room temperature and then at 125 °C for 10 min. The structure of the sensor was fired in a tunnel furnace according to a standard profile. The maximum temperature was 850 °C for 10 min. The thickness of the ZnO layer was about 40 µm.

The surface morphology of our ZnO sensor layer was examined using SEM (Evo LS 15, Zeiss, Oberkochen, Germany).

The crystal structure of the formed ZnO layers was examined by XRD with CuKα radiation (Philips Materials Research Diffractometer, Malvern Panalytical, Malvern, UK). On the basis of characteristic peaks, the average size of crystallites and microstrains in the ZnO structures were determined with the Williamson–Hall (W–H) method [58].

The porosity analysis of obtained sensor layers was performed on the base of Brunauer–Emmett–Teller (BET) multilayer adsorption isotherms. The ZnO structures in powder form were prepared for the tests with the CBD method. The synthesis of these structures was carried out under the same conditions in which they grew directly on alundum substrates. Zinc oxide powders were degassed in a Smart VacPrep (Micromeritics Instrument Corporation, Norcross, GA, USA) preparation station at 200 °C for 6 h. After this time, they were placed in a Micromeritics 3Flex analyzer (Micromeritics Instrument Corporation, Norcross, GA, USA) and adsorption and desorption isotherms at liquid nitrogen temperature were determined. Nitrogen with a purity of 6.0 (as the adsorbate) and helium with a purity of 6.0 (for measuring empty space) were used in the measurements. In addition to the specific BET surface area, pore volume and average pore size were also determined using the Barrett–Joyner–Halend (BJH) method.

The surface chemical compositions of ZnO layers were determined by using XPS (Scienta EW3000, Scienta Omicron GmbH, Taunusstein, Germany). A monochromatized X-ray source based on an Al anode lamp (Al Kα emission line) was used. High-resolution photoelectron energy spectra were recorded using a SCIENTA EW3000 hemispherical analyzer. The pressure in the chamber during measurements was less than 1 × 10^−9^ mbar. To prevent sample charging, a low energy electron flood source was used. The samples were placed on a dedicated sample holder and introduced into the loading chamber when the pressure reached 2 × 10^−8^ mbar. After approx. 1 h, the samples were placed in the XPS chamber and XPS measurement was performed. The CasaXPS software was used for results deconvolution. The concentration of atoms in individual samples was determined on the basis of XPS spectra analysis, taking into account the presence of individual elements O1s, C1s and Zn3s.

PL measurements were performed to determine the impact of the ZnO preparation method on the type and density of defects inside the crystal structure. The measuring system was equipped with a He Cd 325 nm continuous laser, detector (CCD camera) and an optical system. The measurement was made at room temperature in atmospheric air. The signal acquisition time was 1000 min. The final results are based on the five subsequent scans.

The gas-sensitive properties of sensors with various form of ZnO layers were analyzed in ambient air and in an atmosphere containing 2 ppm of chlorine gas. In both atmospheres, the relative humidity was 40%. All measurements were performed in an automated chamber, where it is possible to dose gases from cylinders, permeation tubes and diffusion sources. For chlorine, the permeation tubes were used [59]. The air flow rate was 500 SCCM. As was mentioned above, the change in ZnO layer resistance under the influence of chlorine was determined by the temperature stimulated conductance method (TSC). During these measurements, the current flowing through the sensor material was recorded whereas the temperature was cyclically changed in the range 150–750 °C. The electrodes were polarized with a direct voltage of 2 V for thick layers and 50 mV for microrods. The temperature was changed at a rate of 2 °C/s. The measuring system consisted of a Keithley 2400 (Keithley Instruments Inc., Cleveland, OH, USA) current and voltage source, HP E3632 power supply (Agilent Technologies Inc., Santa Clara, CA, USA), and a galvanostat potentiostat (Solartron SI 1287, Solartron Analytical, Farnborough, UK) [13].

## 3. Results

### 3.1. Structural Characterization

**Figure 2:**
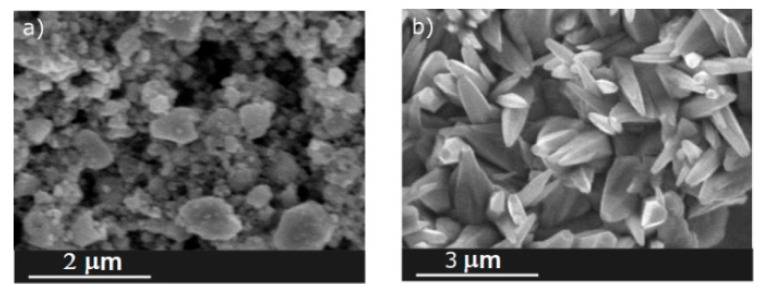
SEM images of ZnO layers formed with the method: (**a**) screen printing; (**b**) chemical bath deposition (CBD).

The morphology of ZnO layers obtained by screen printing and CBD was observed using the SEM method. The microstructure of the obtained oxide layers varied visibly. A thick layer of ZnO, made by screen printing, was porous and made of grains of irregular shapes and various dimensions (Figure 2a). The layer formed as a result of a hydrothermal process was made of pointed q-1D zinc oxide microrods. The side walls of these structures were well formed (Figure 2b).

**Figure 3:**
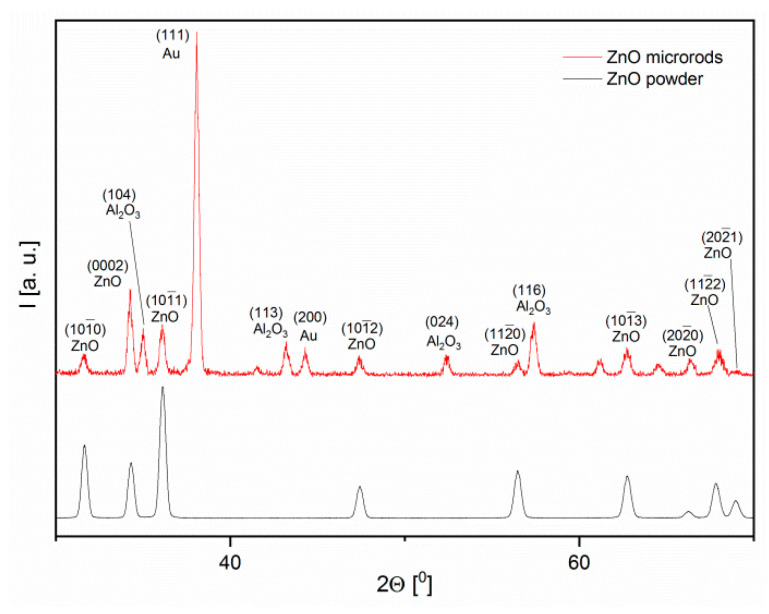
X-ray diffraction patterns of obtained ZnO layers (JCPDS 5-0664).

Based on the XRD diffraction pattern analysis, it was found that both types of layers were made of ZnO with a wurtzite type structure (Zincite, JCPDS 5-0664). Since the layers synthesized by the hydrothermal method were formed directly on the electrode substrate, the X-ray diffraction patterns also show peaks characteristic of gold (electrode) and alundum ceramics (substrate). These peaks do not appear on the zinc oxide diffractograms used in screen printing because ZnO powder was used to form the paste for XRD testing (Figure 3). Before testing, the powder was subjected to the same heat treatment as the printed layers.

The microstrains (*ε*) and the size of crystallites (D) were determined with the Williamson–Hall method (Equation (1)) [58]. It was found that the size of ZnO crystallites formed a thick layer of 22 nm and which is clearly smaller than that of the structures obtained by the hydrothermal method (40 nm). Additionally, the microstrains value is clearly different and amounts to: 5.8 × 10^−4^ for the thick layer and 15.8 × 10^−4^ for q1-D structures [56].

(1) $$\beta_{hkl}cos\left(\Theta \right)=\frac{k\lambda }{D}+4\varepsilon sin\left(\Theta \right)$$

where: *β*—peak half width (FWHM), *Θ*—Bragg angle, *k*—Scherrer constant (0.9), *λ*—wavelength of Cu‑Kα radiation, D—crystallite size and *ε*—lattice strain.

**Figure 4:**
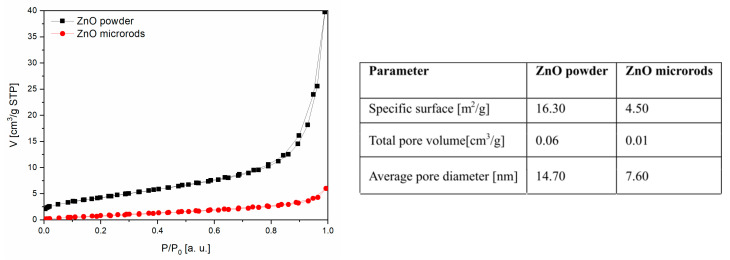
Isotherms of N_2_ adsorption/desorption and Brunauer–Emmett–Teller (BET) analysis results of the samples.

The specific surface area and porosity of the sensor materials were determined with the method of physical nitrogen adsorption based on the adsorption isotherm analysis. Our studies have shown that both sensing materials are mesoporous. The specific surface, pore volume and average pore diameter of the powder used in screen printing are several times bigger than in the structures created in the hydrothermal process (Figure 4).

**Figure 5:**
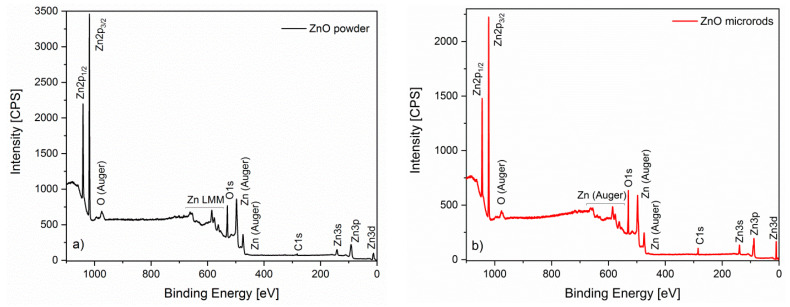
Zinc oxide XPS spectra in the form of: (**a**) powder; (**b**) microrods.

As was mentioned earlier, the surface chemical compositions of both ZnO layers were determined by the XPS method. The spectra of these materials show peaks confirming the presence of elements such as: zinc (Zn2p_1/2_, Zn2p_3/2_, Zn3s, Zn3p, Zn3d), oxygen (O1s) and carbon (C1s). The presence of carbon in the samples indicates the adsorption of organic substances on the surface of our samples or carbon dioxide from the atmosphere (Figure 5). On the basis of our XPS results, it was found that on the surface of microrods the relative concentration of carbon C1s (24.27%) is higher than on the surface of the thick layer (8.77%). This results from the presence of some organic compounds remaining after hydrothermal synthesis. In addition, it was found that for both samples the ratio of Zn/O is about 0.97. Therefore, there is a slightly higher amount of oxygen on the surface compared to zinc, which confirms the phenomenon of oxygen sorption on the surface of the oxide semiconductor.

**Figure 6:**
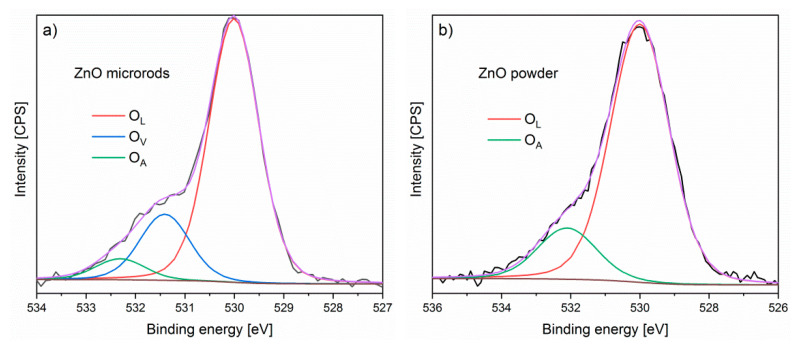
XPS O1s spectral lines after deconvolution of ZnO: (**a**) powder, (**b**) microrods.

The oxygen forms (surface bondings) on the ZnO surface of our both samples were determined on the base of deconvolution of the XPS O1s spectral lines and the relative intensity of the recognized components. For the ZnO microrods, the XPS O1s spectral line consists of three components (Figure 6a). According to literature reports, these peaks are associated with: O^2−^ species in the lattice (O_L_, 530 eV), oxygen vacancies or defects (O_V_, 531.4 eV) and oxygen adsorbed on the surface of ZnO (O_A_, 532.4 eV), as proposed in recent literature papers [35,60,61]. In turn, for the XPS O1s spectral line of ZnO layer made by screen printing, the component corresponding to oxygen vacancies at 531.4 eV was not observed. Furthermore, the two other components of XPS O1s spectral lines have different full with at half maximums (FWHMs) with respect to those observed for ZnO microrods (Figure 6b).

**Figure 7:**
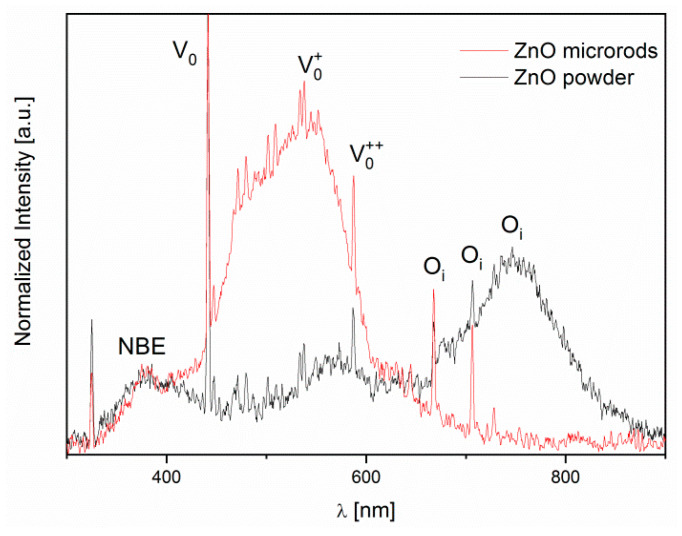
Photoluminescence (PL) spectra of ZnO samples.

As was mentioned above, the photoluminescence (PL) tests were performed to determine the type and concentration of defects occurring in the ZnO crystal structure. Studies have shown the existence of significant differences in the emission bands of the tested oxides synthesized with various methods and, as a consequence, they differ in terms of microstructure. Both in the thick layer spectrum and in the microrod layer, there is an energy maximum around 380 nm associated with the transition of electrons between valence and conductivity bands. Further peaks in the PL spectrum are associated with the presence of certain structural defects in the oxide. In both forms of ZnO, oxygen vacancies and oxygen atoms were found in interstitial positions [62,63]. In the case of microrods, oxygen vacancies are clearly the dominant defect, whereas in the case of ZnO powder, interstitial oxygen is the dominant defect (Figure 7).

### 3.2. Analysis of Gas-Sensitive Properties

Gas-sensitive properties of the obtained ZnO layers in the chlorine atmosphere were determined by the TSC method. Based on these tests, the sensitivity of the sensors was calculated. Sensor sensitivity (S) is the measurement of the sensor response relative to the determined gas and it is defined as a derivative of the processing function after the measured nonelectrical quantity (Equation (2)). Determining the analytical form of this function is extremely difficult. For this reason, the sensitivity of the sensor is commonly defined as the ratio of the sensor signal (conductance) value in the analyzed atmosphere to the value of the signal in the reference atmosphere (Equation (3)). Because chlorine is an oxidizing gas and causes a decrease in the conductivity of the gas-sensitive material, sensitivity was defined as the ratio of the layer conductance value in the reference atmosphere to the conductivity in the atmosphere containing Cl_2_ (Equation (4)) so that the value of sensitivity is greater than 1 [15].

(2) $$S=\frac{df}{dx}$$

(3) $$S_{g}=\frac{G_{gas}}{G_{0}}$$

(4) $$S_{{Cl}_{2}}=\frac{G_{0}}{G_{{Cl}_{2}}}$$

where: *S*—sensitivity, *f*—processing function, *x*—input nonelectrical signal, *S_g_*—gas sensitivity, *G*_0_—layer conductance in the reference atmosphere, *G_gas_*—layer conductance in the atmosphere containing the detecting gas, *S*_*Cl*_2__—chlorine sensitivity and *G*_*Cl*_2__—the conductance of the layer in the atmosphere containing chlorine.

**Figure 8:**
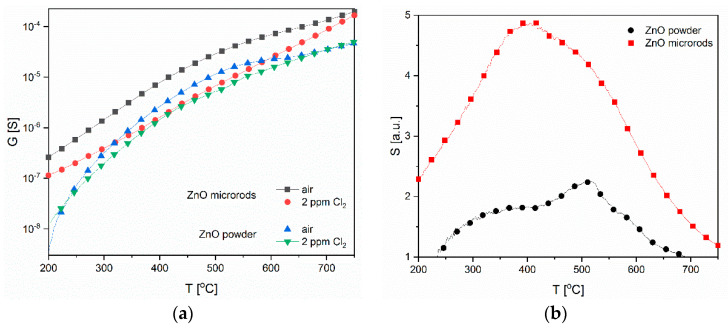
Temperature changes of: (**a**) conductance; (**b**) sensitivity to Cl_2_ of the sensors.

The analysis of obtained results showed that the conductivity of both ZnO layers of various microstructures and other physiochemical properties decreases in the presence of chlorine. This result confirms the oxidative nature of chlorine gas. However, the conductance in the air of the layer made of microrods was definitely higher with respect to the thick layer (Figure 8a). These differences are more clearly visible on the characteristics of S value vs. temperature. The ZnO layer made by screen printing is much less sensitive than the layer made of microrods. In addition, the variation of sensitivity as a function of temperature also differs significantly. They indicate the occurrence of differences in the kinetics of interaction of chlorine with the surface of ZnO layers, which differ in microstructure and the type and concentration of defects (Figure 8b).

## 4. Discussion

In order to explain the differences in behavior of our both ZnO layers, an attempt was made to determine the relationship between the microstructure and ZnO gas-sensitive properties and the mechanism of gas-sensing phenomena occurring on the surface of zinc oxide in the presence of chlorine.

It is well known that some gases interact with the surface of various metal oxides, triggering a change in their conductance (resistance). Depending on the nature of chosen gas and the type of metal oxide conductivity, the resistivity of the gas-sensitive material decreases or increases.

**Figure 9:**
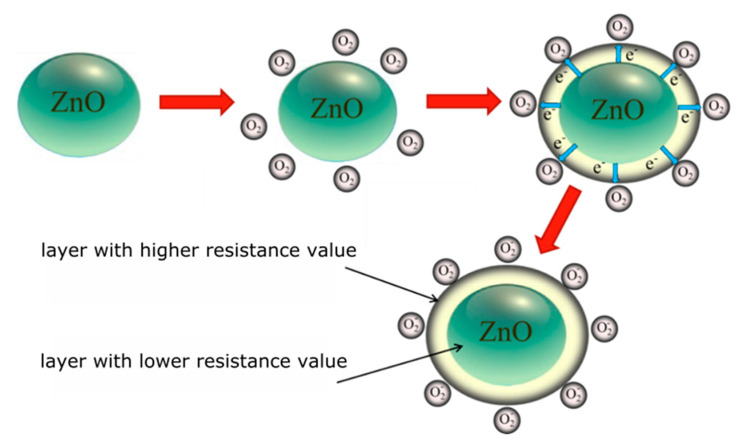
Diagram of the chemical sorption process of oxygen on the surface of an n-type oxide semiconductor.

In the case of n-type semiconductors, e.g., ZnO, their conductivity decreases in the presence of oxidizing gas and increases in the presence of reducing gases. In the reference atmosphere, oxygen is mainly adsorbed on the surface of metal oxide. Oxygen is characterized by high electronegativity and its concentration in the ambient atmosphere is relatively high (about 200,000 ppm). For this reason, it is easily chemisorbed on the surface of metal oxides, in which oxygen vacancies are the dominant defects. The phenomenon occurs at every temperature (T). At higher T values, oxygen undergoes chemisorption and becomes an ion $O_{2}^{-}$, $O_{}^{-}$ or $O_{}^{2-}$ as a result of electron/electrons attachment from the semiconductor conductivity band. Göpel found that the maximum amount of oxygen Θ_max_ (degree of surface coverage) that can undergo chemisorption is less than ≤ 2.5 × 10^−4^ [64]. Because in the chemisorption process of oxygen a charge exchange occurs on the surface of gas-sensitive material, the core-shell structure is created as a result of this process. The outer layer (shell) has a larger and the inside (core) has a smaller resistance (Figure 9). The oxygen–metal interaction is directly related to the width of layer (shell) depleted in electrons and the height of surface potential barrier formed at the grain boundary [22].

When chlorine, which is also an oxidizing gas, appears in the atmosphere surrounding the sensor material, a number of competitive processes take place.

According to literature data, chlorine can form chlorine oxide (Equation (5)), displace chemically adsorbed oxygen (Equation (6)), substitute oxygen in nodal positions $\left(O_{0}^{x}\right)$ (Equation (7)) and fill oxygen gaps $\left(V^{..}\right)$ (Equation (8)). It is believed that all these processes cause a decrease in conductance of the n-type semiconductor gas-sensitive material in the presence of chlorine [46,65].

(5) $$Cl_{2}+2O_{2}\to 2ClO_{2}$$

(6) $$nCl_{2}+O_{2}^{n-}+ne^{-}\to 2nCl^{-}+O_{2}$$

(7) $$Cl_{2}+2O_{0}^{x}+2e^{-}\to 2Cl_{0}^{-}+O_{2}$$

(8) $$Cl_{2}+V_{0}^{..}+4e^{-}\to 2Cl_{0}^{-}$$

The analysis of thermodynamic data rules out the possibility of chlorine oxide formation (Equation (5)) because the Gibbs free energy of formation of these compounds is negative and depending on the degree of chlorine oxidation has values in the range from −18.3 to −63.4 kcal/mol [66]. Moreover, chlorine oxides are not formed as a result of a direct reaction of chlorine with oxygen, chlorine oxides are highly reactive and easily undergo explosive decomposition [66]. As far as chlorine substitution oxygen reactions are concerned (Equations (6) and (7)), the possibility of their occurrence should also be ruled out, since the electronegativity of chlorine is lower than that of oxygen (3.0 < 3.5) and the concentration of oxygen is several orders higher than the concentration of chlorine (200,000 ppm > 2 ppm). Therefore, in the case of chlorine detection, only reactions related to competitive chemical adsorption of chlorine on surface oxygen vacancies should be taken into account (Equation (8)).

In this study, the conditions for the characterization of both ZnO were identical, therefore explanations for the differences in their behaviour in the presence of chlorine gas were attributed to the differences in the structural and chemical properties of the gas-sensitive layer. It is widely believed that the bigger the specific surface of a gas-sensitive material, the greater its sensitivity will be as chemisorption of gases is a process that occurs on the surface of a semiconductor [41]. The analysis of our obtained results does not confirm this claim. However, the sensor layer made of microrods is characterized by a higher sensitivity in a chlorine-containing atmosphere, despite the fact that the specific surface area of the powder is more than three times larger and the pore diameter is almost twice as big than in the case of microrods (Figure 4).

As it is well known, adsorption does not occur on the entire adsorbent surface, but on active centers. For this reason, not only the size of specific surface is important, but also the concentration of individual defects, which usually form active centers.

Han et al. has postulated that semiconductor crystal lattice defects are crucial during oxygen adsorption. As has already been mentioned, during the interaction of this gas with the ZnO surface, an exchange of charge (electrons) occurs. Therefore, it is preferred that the oxide has as many donor levels and as few acceptor levels as possible. Oxygen vacancies are the defects causing an increase in the concentration of donor levels in zinc oxide (Equations (9) and (10)) and interstitial oxygen causes their decrease (Equations (11) and (12)) [67].

(9) $$V_{0}^{x}\to V_{0}^{.}+e^{-}$$

(10) $$V_{0}^{.}\to V_{0}^{..}+e^{-}$$

(11) $$O_{i}^{0}+e^{-}\to O_{i}^{-}$$

(12) $$O_{i}^{-}+e^{-}\to O_{i}^{2-}$$

where: $V_{0}^{x}$—oxygen gap; $V_{0}^{.},\;V_{0}^{..}$—positive charge oxygen gap; $O_{i}^{0}$—oxygen atom in the interstitial position; $O_{i}^{-}$, $O_{i}^{2-}$—negative oxygen ion in the interstitial position.

Thus, the rate of chemisorption process is proportional to the concentration of donor defects (oxygen vacancies), partial pressure of chemisorbed molecules and activation energy (13).

(13) $$v_{ads\;chem}=[V_{0}^{..}]\cdot exp(\frac{E_{a}}{kT})\cdot p_{gas}$$

where: $v_{ads\;chem}$—speed of the chemisorption process, $E_{a}$—activation energy, $p_{gas}$—gas partial pressure, *k*—Boltzmann constant and *T*—temperature.

Because the rate of the chemisorption process, which is associated with the exchange of electric charge, determines the response of the sensor material, the relationship (Equation (13)) shows that if the sensor material contains more vacancies, its response will be greater.

Our XPS and PL studies have shown that ZnO microrods contain significantly more oxygen vacancies than ZnO powder (Figure 6 and Figure 7). For this reason, both the chemical adsorption of oxygen and chlorine should occur more intensively on the surface of microrods. In our studies, we have observed a similar dependency and relationship with ZnO morphology. It was found that the most oxygen vacancies are concentrated on the side planes of the zinc oxide crystal [68,69]. This phenomenon could also explain higher sensitivity value in the case of ZnO microrods than powder in our studies. This is also consistent with Göpel’s claim [64] because achieving a maximum oxygen coverage at the surface will impede, but not reduce, the chlorine chemisorption. This theorem explains the greater sensitivity of ZnO microrods but does not explain the differences in temperature sensitivity changes (Figure 8b).

**Figure 10:**
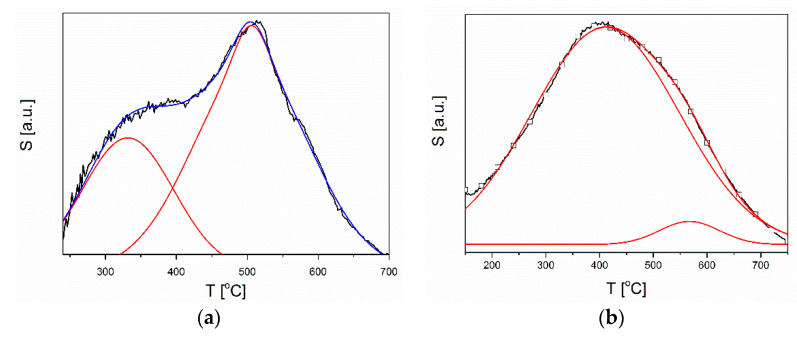
Sensitivity simulation to 2 ppm Cl_2_ as a function of temperature of: (**a**) powder and (**b**) microrods.

As has already been suggested, chlorine and oxygen adsorption are two competitive processes (8, 13), as evidenced by the shape of the characteristics of temperature sensitivity changes. For both our gas-sensitive layers, the S value gradually increased until it reached its maximum value and then decreased. The curves illustrating these changes can be simulated with two peaks illustrating two different processes determining the response of a layer in a chlorine-containing atmosphere (Figure 10). It was assumed that the first peak depends on the intensity of the oxygen interaction with the semiconductor surface. The obtained results were correlated with various physicochemical processes occurring in the ZnO layers in the atmosphere containing oxygen, as described in the literature [64].

According to Göpel, the chemical adsorption of oxygen on the zinc oxide surface begins at 21 °C and reaches its maximum value at 165 °C. At 304 °C, the gas is desorbed and the maximum occurs at 481 °C. However, below this temperature, a sublimation of ZnO begins. If the material is heavily defective, the beginning of sublimation will be found at 360 °C [64].

**Figure 11:**
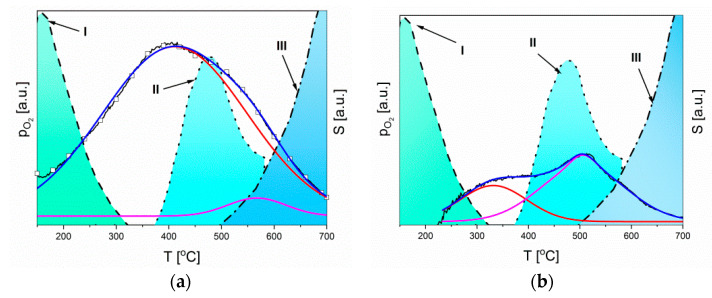
Changes of oxygen partial pressure on the ZnO surface [64] and sensitivity in an atmosphere containing chlorine: (**a**) layer of ZnO microrods, (**b**) layer made by screen printing. I—oxygen chemisorption combined with charge exchange with ZnO. II—desorption of chemisorbed oxygen bound to the ZnO surface. III—sublimation of highly defective zinc oxide.

Comparing the literature data with our obtained temperature changes in sensitivity, it should be stated that when using ZnO layers for the detection of chlorine, the interaction of chlorine with oxygen vacancies and the ZnO sublimation process speed are very important. In the case of both zinc oxide layers, differing in microstructure, when the chemical adsorption rate of oxygen decreases and the desorption process of chemisorbed oxygen begins, the sensitivity increases. This is consistent with the statement that chlorine does not react with chemically adsorbed oxygen (Equation (6)), nor is it able to displace it from the nodal positions (Equation (7)). Therefore, it should be assumed that the first maximum of sensitivity is related to the interaction of chlorine with vacant oxygen vacancies released as a result of oxygen desorption or not occupied by oxygen (Equation (8)). The location of the second peak is probably correlated with the ZnO sublimation process starting at a temperature of about 360 °C and the maximum oxygen desorption at a temperature of about 480 °C. In the sublimation process, the surface layer is removed, so that “new” surface active centres that can interact with chlorine become available (Figure 11).

The higher sensitivity of microrods is primarily the result of an evidently higher concentration of oxygen vacancies, and the slightly distorted peak sensitivity is caused by the slowly starting the sublimation process. This can be demonstrated by the size of the second peak (568 °C), which is definitely smaller than the one corresponding to the first process (408 °C). On this basis, it can be concluded that the factor determining chlorine detection is the concentration of oxygen vacancies and the possibility of chlorine adsorption on them, and to a small extent the ZnO sublimation process (Figure 11a).

In the case of the ZnO thick layer, there is no characteristic clear peak on the temperature dependence of sensitivity, but a very blurred peak. The low concentration of oxygen vacancies in the ZnO layer with such a microstructure causes the sensitivity to be low and increases when the rate of oxygen chemisorption combined with the exchange of charge with ZnO decreases, and then the rate of oxygen desorption increases. However, the sensitivity of this sample reaches the highest value when oxygen desorption occurs at a maximum speed and the sublimation process is already clearly taking place. As has already been stated, chlorine adsorption depends on the concentration of active centers not occupied by adsorbed oxygen. In the case of the fine-crystalline thick layer, the initial concentration of oxygen vacancies is low, and their availability for chlorine increases only as a result of O_2_ desorption and ZnO sublimation. For this reason, in this case, the second peak associated with both ZnO sublimation and maximum oxygen desorption dominates (Figure 11b).

**Figure 12:**
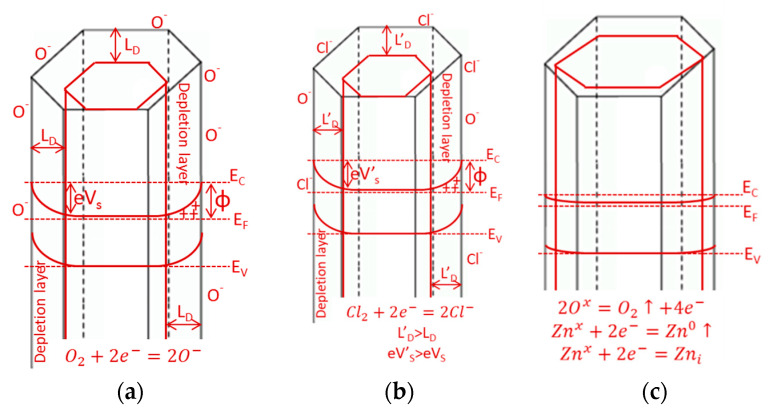
Scheme of chlorine detection steps on ZnO surfaces: (**a**) O_2_ chemisorption, (**b**) partial O_2_ desorption and Cl_2_ adsorption, (**c**) sublimation of ZnO. L_D_—Debye length, E_C_—Conduction band energy, E_F_—Fermi level and E_V_—Valence band energy.

Summarizing, it can be concluded that the detection of chlorine on the ZnO surface is strongly correlated with the structure of ZnO and the operating temperature of the sensor. There are three main processes. By analyzing them with increasing temperature, we determined that the first adsorption of oxygen takes place on the semiconductor surface, which increases the width of the depleted layer (Figure 12a). This process requires the same active species as chlorine adsorption (oxygen vacancies). Given the slower O_2_ chemisorption and the faster O_2_ desorption (Figure 11), the more chlorine molecules can become trapped on the ZnO surface. This process also increases the width of the depleted layer (Figure 12b). At least semiconductor sublimation is taking place. First, it created “new” surface active centers that can interact with chlorine and then, after further temperature increases, all gas molecules desorption occurs what causes the reduction in the width of the depleted layer (Figure 12c).

## 5. Conclusions

In the presented article, two ZnO layers of different microstructures and chemical properties were examined. A first microcrystalline thick ZnO layer was made by screen printing from ZnO powder obtained in a coprecipitation reaction. A second ZnO layer made of microrods (one-dimensional structures) were synthesized by the chemical bath deposition technique. The crystal structure of both ZnO layers was a typical wurtzite type. The average size of one-dimensional ZnO crystallite structure was bigger than that of the crystallites from which the thick layer was built. The specific surface and porosity of the thick ZnO layer were several times bigger than the ZnO layer made of microrods. However, a ZnO layer made of microrods manifested a greater value of sensitivity in the presence of chlorine than a fine-crystal one made by screen printing. The obtained results are in a contradiction to the generally accepted views on the direct correlation between sensitivity and the size of active surface. The thick ZnO layer characterized by an almost four times bigger specific surface area than microrods exhibited a twice as low sensitivity. This is probably related to the differences in microstructure and differences in the concentration of oxygen vacancies. The comparative analysis of literature data and our results showed that the main parameter determining the sensitivity of chlorine gas detection is the concentration of donor levels (oxygen vacancies) related to the ZnO microstructure. Both the concentration of oxygen vacancies and the microstructure of this ZnO layers depend on their synthesis method. The photoluminescence tests have clearly shown that, in microrods, donor defects, i.e., oxygen vacancies, are the dominant defects. In the granular structure of the thick ZnO layer, the dominant defects are interstitial oxygen. It has been shown that, in a chlorine-containing atmosphere, this gas interacts with the free surface vacancies of ZnO. This reaction is a competitive process for the chemical sorption of oxygen at the surface oxygen vacancies. However, the concentration of surface vacancies in ZnO is not constant. At a temperature higher than 350 °C as the sublimation of oxide begins, with the rate depending on the ZnO microstructure. As a result of the sublimation effect, the new surface vacancies revealed that they are not filled with oxygen, which can be occupied by chlorine. Based on the results of our performed studies, it can be stated that the physicochemical processes occurring on the surface of gas-sensitive materials depend not only on such basic material parameters as the specific surface and porosity, but also on the degree and type of defects, the properties of chosen gas and the specific properties of gas-sensitive material such as temperature and the speed of sublimation. Both the degree and type of defects can be modified by using the various ZnO morphological forms synthesized by different methods. As a consequence, the sublimation rate change can also be obtained.
